# Supplementary material for: Topoisomerase I (TOP1) dynamics: conformational transition from open to closed states
Source: Nat Commun. 2022 Jan 10;13:59. doi: 10.1038/s41467-021-27686-7 (PMC8748870; doi:10.1038/s41467-021-27686-7)
Supplement: Supplementary file 1 — Supplementary information [file 41467_2021_27686_MOESM1_ESM.pdf]

Supplementary Table 1: Codon-optimized genes

Sequence of the codon-optimized genes used in this study. The sequences of the His-tag and the cleavage site (blue) and the sequence of the gene (black) are shown.

Codon-Optimised gene	Sequence
CsTop1	ATGAAACATCACCATCACCATCACCCCATGAGCGATTACGACATCCCCACTACTGAGAATCTTTATTTTCAGGGC<br>GCCATGGTGAAATGGCGTACCCTGGTCCACAATGGTGTCGCACTGCCGCCGCCGTATCAACCGAAAGGTCTGTCA<br>ATCAAAATCCGTGGTGAAACCGTGAAACTGGATCCGCTGCAGGAAGAAATGGCGTATGCCTGGGCACTGAAGAAA<br>GATACGCCGTACGTGCAGGATCCGGTTTTTCAGAAAAACTTTCTGACCGATTTTCTGAAAACGTTCAACGGCCGC<br>TTTCAGGACGTTACCATCAACGAAATCGATTTCAGCGAAGTCTACGAATACGTGGAACGTGAACGCCAACTGAAA<br>GCGGATAAAGAATACCGTAAGAAAATTTCTGCCGAACGTAAACGCCTGCGTGAAGAACTGAAAGCGCGCTACGGC<br>TGGGCCGAAATGGATGGTAAACGTTTCGAAATTGCGAACTGGATGGTTGAACCGCCGGGCATCTTTATGGGTCGT<br>GGTAATCATCCGCTGCGTGGTCGTTGGAAACCGCGTGTCTATGAAGAAGACATTACCCTGAATCTGGGTGAAGAT<br>GCGCCGGTCCCGCCGGGTAATTGGGGTCAGATCGTGCATGATCACGACTCAATGTGGCTGGCCCGCTGGGATGAC<br>AAACTGACCGGTAAAGAAAAATATGTGTGGCTGAGCGATACCGCAGATATTAAACAGAAACGCGACAAAAGCAAA<br>TACGACAAAGCAGAAATGCTGGAAAACCATATTGATCGTGTGCGCGAGAAAATCTTTAAAGGTCTGCGTAGCAAA<br>GAACCGAAAATGCGTGAAATTGCACTGGCCTGTTATCTGATTGATCGTCTGGCAATGCGTGTTGGTGATGAAAAA<br>GATCCGGATGAAGCAGATACCGTTGGTGCAACCACCCTGCGTGTTGAACATGTTAAACTGCTGGAAGATCGCATC<br>GAGTTTGATTTTCTGGGTAAAGATAGCGTTCGTTGGCAGAAAAGCATTGATCTGCGTAATGAACCTCCGGAAGTT<br>CGTCAGGTTTTTGAAGAACTGCTGGAAGGTAAAAAAGAAGGCGATCAGATTTTCCAGAACATCAATAGCCGTCAT<br>GTGAATCGTTTTCTGGGCAAAATTGTTAAAGGCCTGACCGCAAAAGTGTTTCGTACCTATATCGCAACCAAAATC<br>GTCAAAGATTTCCTGGCAGCAATTCCGCGTGAAAAAGTTACCAGCCAAGAAAAATTCATCTATTACGCCAAACTG<br>GCCAATCTGAAAGCAGCAGAAGCACTGAATCATAAACGTGCACCGCCTAAAAATTGGGAACAGAGCATCCAGAAA<br>AAAGAGGAACGCGTTAAAAAACTGATGCAGCAGCTGCGCGAAGCCGAAAGCGAGAAAAAAAAAGCACGTATTGCA<br>GAACGTCTGGAAAAAGCAGAACTGAACCTGGATCTGGCAGTTAAAGTTCGTGATTACAATCTGGCGACCAGCCTG<br>CGTAACTATATTGATCCGCGTGTGTATAAAGCATGGGGTCGTTATACCGGTTATGAATGGCGTAAAATCTATACC<br>GCAAGCCTGCTGCGTAAATTCAAATGGGTTGAAAAAGCCAGCGTTAAACATGTGCTGCAGTATTTTGCCGAAAAA<br>CTGGCAAAAGATGTGGATAAAGGTATGCAGGTTAAAGCAGCCGTTTAA
$CsTOP1^{HsLinker\text{-}Cter}$	ATGAAACATCACCATCACCATCACCCCATGAGCGATTACGACATCCCCACTACTGAGAATCTTTATTTTCAGGGC<br>GCCGTGAAATGGCGTACCCTGGTCCACAATGGTGTCGCACTGCCGCCGCCGTATCAACCGAAAGGTCTGTCAATC<br>AAAATCCGTGGTGAAACCGTGAAACTGGATCCGCTGCAGGAAGAAATGGCGTATGCCTGGGCACTGAAGAAAGAT<br>ACGCCGTACGTGCAGGATCCGGTTTTTCAGAAAAACTTTCTGACCGATTTTCTGAAAACGTTCAACGGCCGCTTT<br>CAGGACGTTACCATCAACGAAATCGATTTCAGCGAAGTCTACGAATACGTGGAACGTGAACGCCAACTGAAAGCG<br>GATAAAGAATACCGTAAGAAAATTTCTGCCGAACGTAAACGCCTGCGTGAAGAACTGAAAGCGCGCTACGGCTGG<br>GCCGAAATGGATGGTAAACGTTTCGAAATTGCGAACTGGATGGTTGAACCGCCGGGCATCTTTATGGGTCGTGGT<br>AATCATCCGCTGCGTGGTCGTTGGAAACCGCGTGTCTATGAAGAAGACATTACCCTGAATCTGGGTGAAGATGCG<br>CCGGTCCCGCCGGGTAATTGGGGTCAGATCGTGCATGATCACGACTCAATGTGGCTGGCCCGCTGGGATGACAAA<br>CTGACCGGTAAAGAAAAATATGTGTGGCTGAGTGATACGGCAGACATCAAACAGAAACGTGATAAATCCAAATAC<br>GACAAAGCTGAAATGCTGGAAAATCATATTGATCGCGTTCGTGAAAAAATCTTCAAAGGCCTGCGCTCGAAAGAA<br>CCGAAAATGCGTGAAATTGCTCTGGCGTGCTACCTGATCGACCGCCTGGCGATGCGTGTTGGCGATGAAAAAGAT<br>CCGGACGAAGCAGACACCGTCGGTGCTACCACGCTGCGCGTCGAACACGTGAAACTGCTGGAAGATCGTATTGAA<br>TTTGACTTCCTGGGTAAAGATAGCGTGCGCTGGCAGAAATCTATCGATCTGCGCAACGAACCGCCGGAAGTTCGT<br>CAAGTCTTTGAAGAACTGCTGGAAGGCAAGAAAGAAGGCGATCAGATTTTCCAAAACATCAATAGTCGCCATGTT<br>AATCGTTTTCTGGGCAAAATTGTTAAAGGTCTGACCGCAAAAGTCTTCCGCACCTACATCGCTACGAAAATCGTG<br>AAAGATTTTCTGGCGGCCATTCCGCGTGAAAAAGTTACGAGTCAGGAAAAATTCATCTATTACGCAAAACTGGCT<br>AACCTGAAAGCAGCTGAAGCGCTGAATCACCAGCGCGCACCGCCGAAAACCTTTGAAAAATCCATGATGAACCTG<br>CAGACGAAAATCGATGCGAAGAAAGAACAACTGGCCGATGCACGTCGCGACCTGAAATCAGCTAAAGCGGACGCC<br>AAAGTGATGAAAGATGCGAAAACCAAGAAAGTGGTTGAAAGCAAAAAGAAAGCGGTCCAGCGCCTGGAAGAACAA<br>CTGATGAAACTGGAAGTGCAGGCAACGGATCGTGAAGAAAACAAACAAATTGCTCTGGGCACCTCTAAACTGAAT<br>TATCTGGACCCGCGTATCACGGTGGCCTGGTGTAAAAAATGGGGTGTTCCGATTGAAAAAATCTACAATAAAACG<br>CAGCGTGAAAAATTTGCTTGGGCTATTGATATGGCGGATGAAGACTATGAATTCTAA

Supplementary Table 2: primer sequences

Sequence of the DNA oligonucleotides used in this study.

Name	Sequence	Construct
CsFL154R	5’-TAATCATCCGAAGCGTGGTCGTTG-3’	L154K point mutation in CsTop1
CsRL154R	5’-CCACGACCCATAAAGATG-3’	
CsFL399RL402R	5'-CGCTTCAGCTGCTTTCCGGTTAGCCCGTTTTGCGTAATAGAT-3'	L399R L402R point mutations in CsTop1
CsRFL399L402R	5'-ATCTATTACGCAAAACGGGCTAACCGGAAAGCAGCTGAAGCG-3'	
CsFHsCat	5’ CTACTGGATCCGCGTGTGTATAAAG-3’	CsTOP1^{HsTyr} point mutations
CRFHsCat	5’ TTCAGTTTGCTGGTCGCCAAGATTGTA-3’	
Q5SDM_12/7/2016_F	5’-GAAATGGTGGGAAGAAGAGCGCTATCCTGAAGGCATCGTGAAATGGCGTACCCTG-3’	CsTOP1^{HsNter} insertion
Q5SDM_12/7/2016_R	5’- CACTTCTGTTCCTCTTCTTTCTTCGGCTTCTTTTTCTTGTGATGGTGATGGTGATG-3’	
Q5SDM_4/13/2019_F	5’-CCCTAGCGCAGATATTAAACAGAAACG-3’	Hinge-1 point mutations (MLNPS)
Q5SDM_4/13/2019_R	5’-TTCAGCATCACATATTTTTCTTTACCGG-3’	
Q5SDM_4/13/2019_F	5’-CCTCCCGCAGATATTAAACAGAAACG-3’	Hinge-2 point mutations (WLSPP)
Q5SDM_4/13/2019_R	5’-GCTCAGCCACACATATTTTTCTTTACC-3’	
CsTop1WLSGGfwd	5’- AAAAATATGTGTGGCTGAGCGGTGGCGCAGATATTAAACAGAAACG-3’	Hinge-3 point mutations (WLSGG)
CsTop1WLSGGrev	5’- CGTTTCTGTTTAATATCTGCGCCACCGCTCAGCCACACATATTTTT-3’	
CsTop1Y207Afwd	5’- CTGACCGGTAAAGAAAAAGCTGTGTGGCTGAGCGATAC-3’	Y207A point mutation in CsTop1
CsTop1Y207Arev	5’-GTATCGCTCAGCCACACAGCTTTTTCTTTACCGGTCAG-3’	

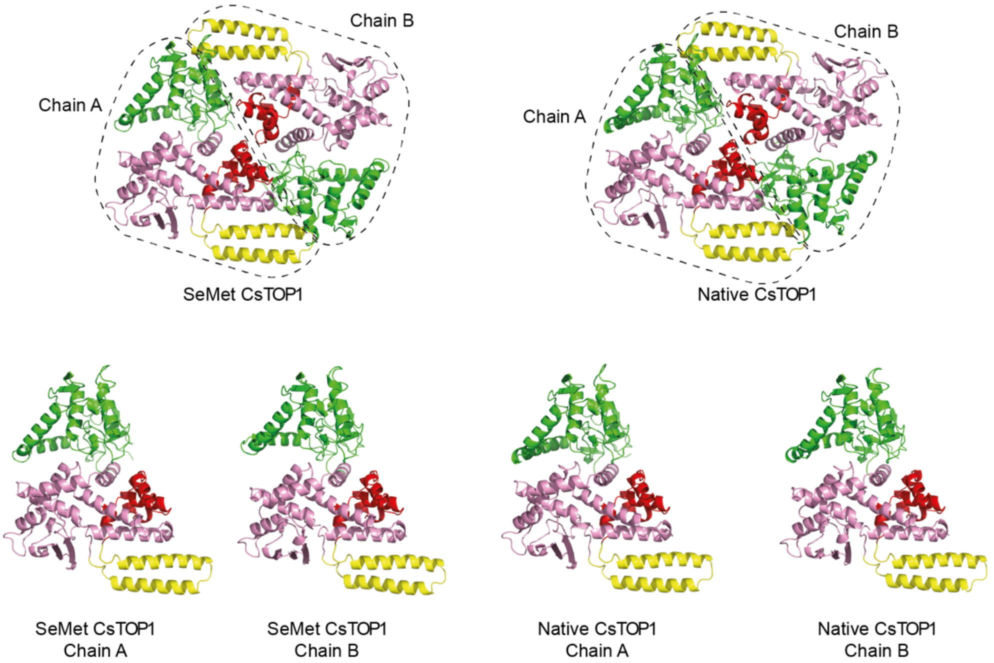

Supplementary Figure 1: CsTOP1 structure in one asymmetric unit.
Cartoon representation of the two CsTOP1 chains found in one asymmetric unit in SeMet CsTOP1 and native CsTOP1 crystals. The different subunits were further superposed. Coloring scheme is the same as Fig. 1.

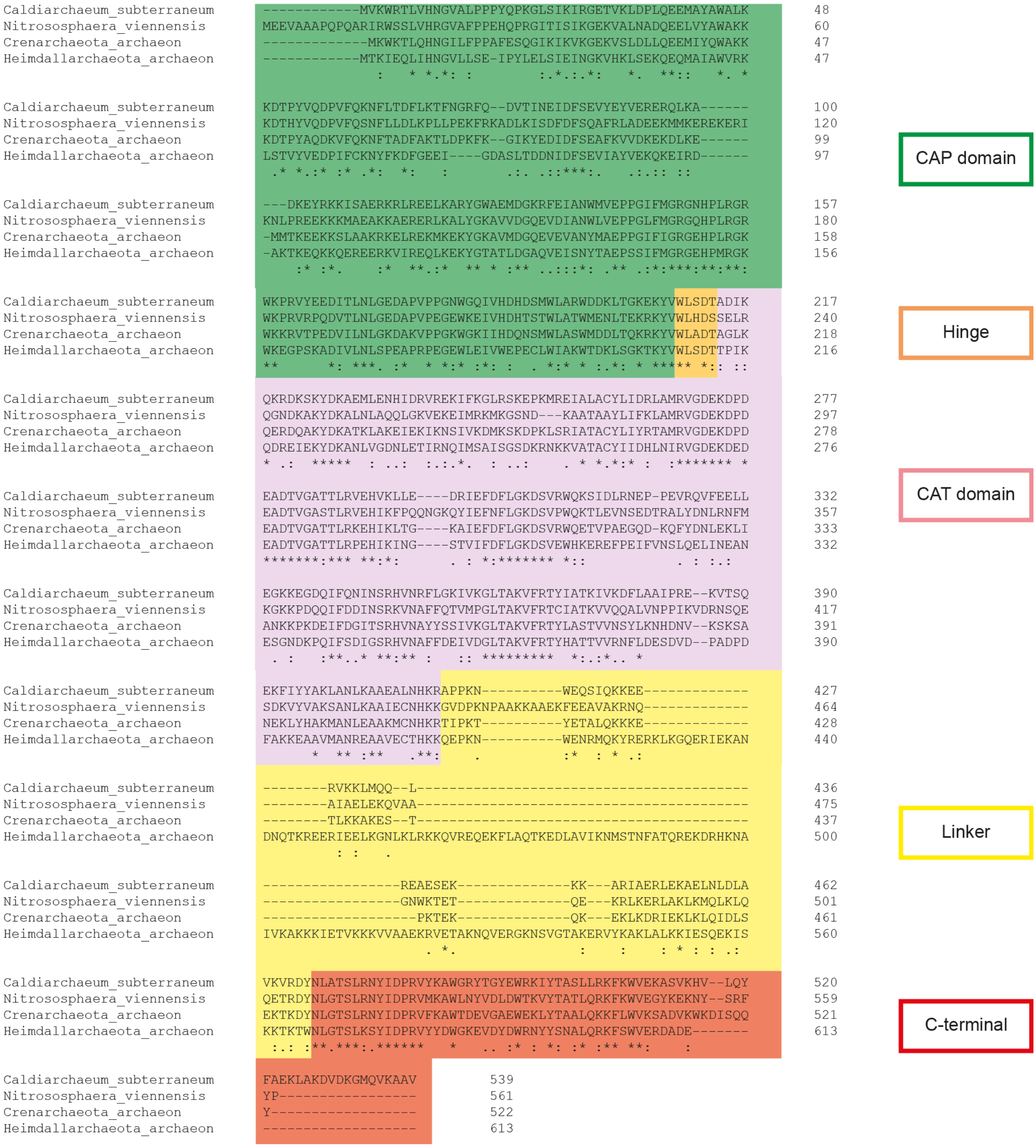

Supplementary Figure 2: Alignment of archaeal TOP1 sequences.
Alignment of four archaeal TOP1 sequences from different phyla including *Caldiarchaeum subtarreneum* (Aigarchaeota), *Nitrososphaera viennensis* (Thaumarchaeota), *Crenarchaeota* archaeon (Crenarchaeota) and *Heimdallarchaeota* archaeon (Heimdallarchaeota, Asgard).

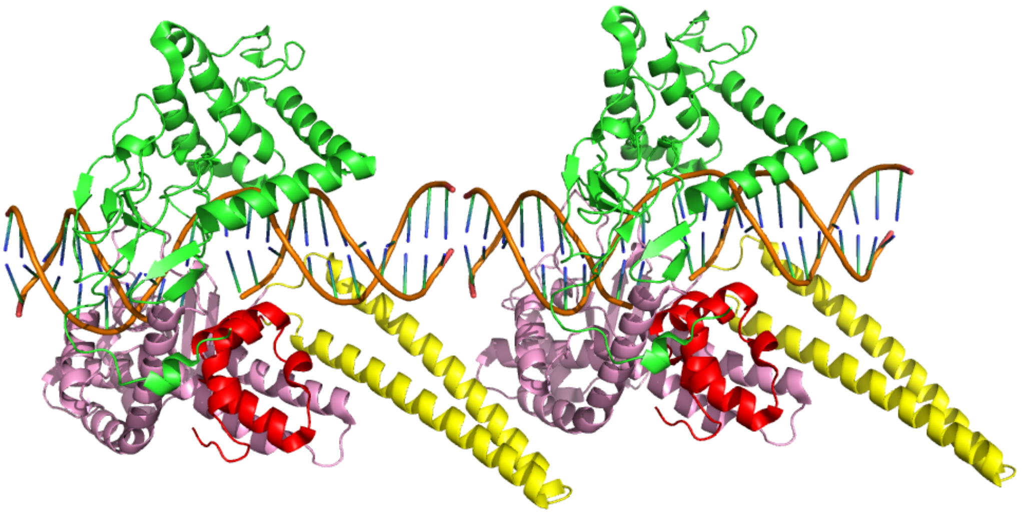

Supplementary Figure 3: Packing artifact of HsTOP1-DNA.
Cartoon representation of two complexes within the packing of HsTop1-DNA crystal structure (PDB 1K4T). The linker domain (yellow) of one TOP1 is pushed away from the DNA by the CAT domain (pink) of a neighboring TOP1 molecule. Coloring scheme is the same as Fig. 1.

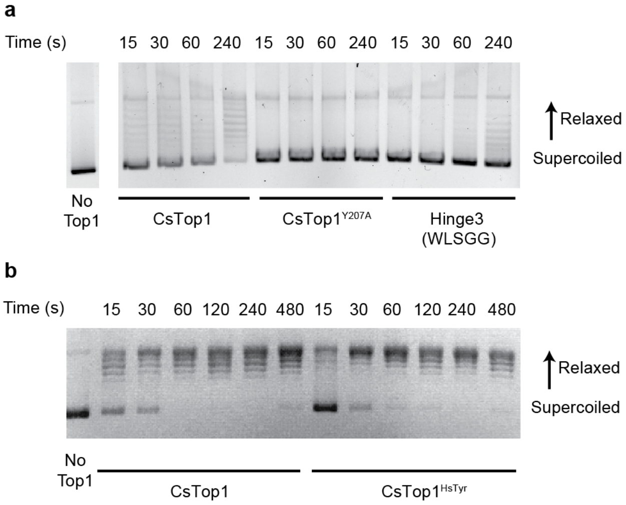

Supplementary Figure 4: Relaxation of supercoiled DNA by mutated CsTop1.
a) Relaxation assay with CsTop1, CsTop1(Y207A) and CsTop1 (Hinge3, WLSGG). **b)** Relaxation assay with CsTop1 and CsTop1(HsTyr). The kinetics of relaxation is shown for each construct. Both experiments were done twice independently.
